# Association of receiving opioid medication-assisted treatment with sexual identity and mental health/substance use disorder symptoms in a nationally representative sample of adults

**DOI:** 10.21203/rs.3.rs-2837899/v1

**Published:** 2023-04-24

**Authors:** David Adzrago, Gabrielle S Evans, Emanuelle M Dias, Victoria Kwentua, Grace Elizabeth White, J. Michael Wilkerson

**Affiliations:** The University of Texas Health Science Center at Houston (UTHealth); The University of Texas Health Science Center at Houston (UTHealth); The University of Texas Health Science Center at Houston (UTHealth); The University of Texas Health Science Center at Houston (UTHealth); The University of Texas Health Science Center at Houston (UTHealth); The University of Texas Health Science Center at Houston (UTHealth)

**Keywords:** Opioid use, Opioid medication-assisted treatment, Sexual minority, Depression, substance use disorder

## Abstract

**Background:**

Although the literature suggests that medication-assisted treatment (MAT) is an effective treatment for opioid use disorder, limited studies have assessed the prevalence or the association between MAT use and sexual identity, mental health, or substance use disorder among a nationally representative sample. We assessed the prevalence and association of opioid MAT use between sexual identity, depressive disorder symptoms, alcohol use dependence, and marijuana use dependence in the United States.

**Methods:**

We used the 2019 National Survey on Drug Use and Health public-use data on adults aged 18–64 years (*N* = 38,841) to conduct a weighted multivariable logistic regression analysis.

**Results:**

A total of 4.80% and 2.32% of the population identified as bisexual and lesbian/gay, respectively. About 0.31% (612,750 people) of the population reported receiving opioid MAT, 3.73% had alcohol use dependence, 1.42% had marijuana use dependence, and 9.13% had major depressive episode (MDE) symptoms. Of those who had received opioid MAT, 0.57% were bisexuals and 1.07% were lesbians/gays, 0.65% were people with alcohol use dependence, 2.32% with marijuana use dependence, and 1.59% with MDE symptoms. Lesbian/gay individuals were more likely to receive opioid MAT (AOR = 3.43, 95% CI = 1.42, 8.25) compared to heterosexual individuals. The odds were higher for people with marijuana use dependence (AOR = 3.44, 95% CI = 1.47, 8.06) and MDE symptoms (AOR = 5.22, 95% CI = 3.46, 7.89) than their counterparts.

**Conclusions:**

In this study, sexual minorities, people with MDE symptoms, and those dependent on marijuana use were more likely to receive opioid MAT, suggesting the need to investigate further opioid use disorder symptoms and their risk factors among these populations.

## Background

In 2020, 9.5 million of the United States (U.S.) population over the age of 12 years reported misusing opioids in the past year [1]. Opioids are a class of drugs composed of legally prescribed analgesics (e.g., oxycodone, hydrocodone, morphine) and illegal substances (e.g., heroin, synthetic opioids) [2, 3]. Given opioids’ effectiveness in treating pain, opioids are most often prescribed to patients by their physicians [4, 5]. However, opioid abuse often leads to a chronic lifelong disorder known as opioid use disorder (OUD) [6]. OUD is defined by the American Psychiatric Association [7] as a pattern of inappropriate use of opioids, either prescribed or illegal, resulting in two or more criteria that reflect impaired health or function occurring within one year. Examples of criteria include taking larger amounts of opioids over a longer period than intended, craving or having a strong desire to use opioids, or giving up activities because of opioid use [8]. About 2.1 million people are living with OUD in the U.S., and the opioid crisis was declared a public health emergency in October 2017 [7, 9].

The prevalence of OUD is disproportionately higher in certain groups, including sexual and gender minority persons. Across all age cohorts, sexual and gender minority persons have a significantly higher risk of experiencing an OUD. The risk of having an OUD diagnosis is almost three times higher than among their heterosexual counterparts [10–13].

Persons with an OUD diagnosis are more likely to have certain comorbid diagnoses. For example, approximately one-third of persons with an OUD also have a depressive disorder [14]. Individuals experiencing severe pain are more likely to be prescribed opioids, which, when combined with antidepressants to treat co-occurring depressive symptoms, increase the risk of unintentional overdose [15]. Persons with an OUD are 8.4 times more likely to have an alcohol use disorder than those without an OUD [16]. Significantly, having a comorbid OUD and alcohol use disorder increases the risk of unintentional overdose due to a bi-directional relationship between the two [17–20]. A similar pattern has been identified among persons with comorbid OUD and marijuana use disorders [21].

Medication-assisted treatment (MAT) has been proven to be an effective treatment for those with OUD [8, 22]. MAT is the use of medications, often combined with behavioral therapy, to treat persons in recovery with a substance use disorder. MAT has been shown effective in helping reduce opioid use and the risks associated with the misuse. An emerging body of research also demonstrates that the integration of MAT into treatment and recovery support services increases client retention [8, 23, 24].

Although there is evidence that MAT is an effective treatment for persons with an OUD, there is a need to estimate the prevalence and the association between opioid MAT use and sexual identity, mental health, OUD, or substance abuse among a nationally represented sample [24]. Obtaining prevalence estimates and the associations will help understand the distribution and potential risk factors of MAT use in the population subgroups and areas to expand MAT use interventions to reduce the burdens of OUD in the population. Therefore, this analysis aimed to: (1) estimate the prevalence of opioid MAT among persons who identify as a sexual minority or self-reported experiencing depressive symptoms, alcohol use dependence, and marijuana use dependence; and (2) obtain parameter estimates that approximate the strength of the associations between opioid MAT use with sexual identity, depressive disorder symptoms, alcohol use dependence, and marijuana use dependence.

## Methods

### Study design

We used de-identified public-use data from the 2019 National Survey on Drug Use and Health (NSDUH) for this study. Details of the construction of the samples, survey questions, and survey administration can be found in the Center for Behavioral Health Statistics and Quality [25] and Substance Abuse and Mental Health Services Administration (SAMHSA) [26]. The NSDUH is an annual cross-sectional survey in the U.S that uses a complex, multistage area probability sample of the U.S. civilian, noninstitutionalized population in each of the 50 states and the District of Columbia. This survey assesses substance use and mental health among the population. The data consists of a total of 56,136 individuals aged 12 years and older. We, however, conducted our analysis on adults aged 18 years to 64 years of age, which consists of a total of 38,841 adults with complete data on receiving opioid medication-assisted treatment (MAT) status. Only adults aged 18–64 years were considered in our analysis because individuals aged 12–17 years or 65-plus years had no MAT use prevalence in the dataset.

### Measures

#### Dependent variable

The dependent variable is receiving opioid MAT status, which was self-reported. The participants were asked to indicate whether they had received opioid MAT in the past year (yes/no).

#### Independent variables

We examined four main independent variables: 1) sexual identity, 2) major depressive episode (MDE) symptoms in the past year, 3) past-year alcohol use dependence, and 4) past-year marijuana use dependence. Sexual identity was measured by asking the participants to self-report their sexual identity using heterosexual, lesbian, gay, or bisexual response options. Our analysis categorized the sexual identity responses as heterosexual, lesbian/gay, or bisexual. Due to the limited sample size within groups, we combined lesbian/gay and bisexual participants into one category in Table 3.

MDE symptom status was determined in the NSDUH based on the Diagnostic and Statistical Manual of Mental Disorders 5th edition (DSM-5) [7, 27]. MDE questions measured if participants had experienced an MDE in the past year (see details in Center for Behavioral Health Statistics and Quality [25]). Based on the self-report on the DSM-5 questions, the participant was classified by the NSDUH as having MDE in the past year if the participant had a lifetime MDE and a time in the past 12 months when the participant felt depressed or lost interest or pleasure in daily activities for two weeks or longer, while also having some of the other symptoms for lifetime MDE. Otherwise, the participant was classified by the NSDUH as not having MDE in the past year if the participant had no lifetime MDE or with lifetime MDE but no period of depression lasting two weeks or longer while having other symptoms for lifetime MDE.

Alcohol use dependence was determined with questions based on DSM-4 criteria [26, 28]. A participant was classified with alcohol use dependence if the participant met three or more of the seven DSM-4 alcohol use dependence criteria. Else, the participant was classified as not having alcohol use dependence. These criteria include if the participant: 1) spent a great deal of time over a month or more getting, using, or getting over the effects of alcohol, 2) used alcohol more often than intended or was unable to keep set limits on alcohol use, 3) needed to use alcohol more than before to get desired effects or noticed that same amount of alcohol use had less effect than before, 4) inability to cut down or stop using alcohol every time tried or wanted to, 5) continued to use alcohol even though it was causing problems with emotions, nerves, mental health, or physical problems, 6) alcohol use reduced or eliminated involvement or participation in important activities, and 7) reported experiencing two or more alcohol withdrawal symptoms at the same time that lasted longer than a day after alcohol use was cut back or stopped.

Marijuana use dependence was assessed based on six DSM-4 marijuana use dependence criteria [26, 28]. A participant was classified with marijuana use dependence if they met three or more of the six criteria. Otherwise, the participant was classified as not having marijuana use dependence. The criteria are: 1) Spent a great deal of time over a month or more getting, using, or getting over the effects of marijuana, 2) Used marijuana more often than intended or was unable to keep set limits on marijuana use, 3) Needed to use marijuana more than before to get desired effects or noticed that same amount of marijuana use had less effect than before, 4) Inability to cut down or stop using marijuana every time tried or wanted to, 5) Continued to use marijuana even though it was causing problems with emotions, nerves, mental health, or physical problems, and 6) Marijuana use reduced or eliminated involvement or participation in important activities.

### Covariates

Our analyses adjusted for sociodemographic characteristics based on previous studies [10–13]. These variables include age (18–25, 26–34, 35–49, 50–64), sex (male/female), race/ethnicity (non-Hispanic White, non-Hispanic Black/African American, Hispanic, and Other race [non-Hispanic Native American/Alaskan Native, non-Hispanic Asian American, non-Hispanic Native Hawaiian/Other Pacific Islander, and non-Hispanic more than one race]), level of education completed (Twelfth grade or less, High School diploma/GED, some college credit but no degree, Associate's degree, and college graduate or higher), total family income (<$20,000; $20,000 to $49,999; $50,000–$74,999; and ≥ $75,000), and employment status (employed full time, part-time, unemployed, or other [i.e., students, persons keeping the house or caring for children full time, retired or disabled persons, or other persons not in the labor force]). These variables were analyzed as categorical variables in our study.

### Statistical Analyses

The data analysis was performed using STATA/SE, version 16.1 [29]. Descriptive analyses were performed to describe percentages of the participants’ sociodemographic characteristics, MDE symptoms, alcohol use dependence, and marijuana use dependence by the status of receiving opioid MAT (see Table 1). We computed bivariate analyses to assess the association between receiving opioid MAT status and sociodemographic characteristics, sexual identity, MDE symptoms, alcohol use dependence, and marijuana use dependence, respectively, using chi-square tests. Furthermore, we conducted a multivariable logistic regression analysis to examine the association between receiving opioid MAT and sexual identity, MDE symptoms, alcohol use dependence, and marijuana use dependence, adjusting for the sociodemographic variables (see Table 2). We examined the association between receiving opioid MAT and sexual identity, MDE symptoms, alcohol use dependence, and marijuana use dependence, adjusting for the sociodemographic variables, among individuals diagnosed with opioid use disorder (OUD) symptoms using multivariable logistic regression analysis (see Table 3). We reported adjusted odds ratios (AORs) and 95% confidence intervals (95% CIs) and considered statistically significant results at p < 0.05 using the Wald test or Wald Chi-Squared Test.

All the analyses, except the frequencies, were weighted using the NSDUH survey weight to obtain nationally representative estimates. The NSDUH survey weight helps obtain weighting and clustering effects such as the unequal probability of sampling, non-response, and post-stratification adjustments [25]. The NSDUH nesting variables were also used to capture explicit stratification, ascertain clustering with the data, and obtain accurate variance estimates [25]. There were 509 (unweighted = 1.31% and weighted = 1.24%) and 826 (unweighted = 2.13% and weighted = 2.25%) missing observations on MDE and sexual identity, respectively. However, this missingness is insignificant and negligible because it is less than 10% to impact our findings [30, 31]. Multicollinearity among the independent variables was also examined using the variance inflation factor (VIF). No significant multicollinearity was found because the mean VIF value was 1.10 [32].

## Results

### Descriptive and bivariate statistics

The descriptive and bivariate statistics of the study sample (Weighted N = 197,554,210 and Unweighted N = 38,841) are presented in Table 1. The majority of the population was aged 50–64 (31.68%), female (50.83%), non-Hispanic Whites (59.66%), had college graduate or higher degrees (32.89%), had $75,000 or more total annual family income (39.64%), and employed full time (59.94%). A total of 4.80% and 2.32% of the population identified as bisexual and lesbians/gay, respectively. Overall, 3.73% had alcohol use dependence, 1.42% had marijuana use dependence, and 0.31% (representing 612,750 population) reported receiving opioid medication-assisted treatment (MAT). The bivariate analysis results showed that age (p < 0.001), race/ethnicity (p = 0.004), sexual identity (p = 0.002), level of education completed (p < 0.001), total annual family income (p < 0.001), employment status (p < 0.001), alcohol use dependence (p = 0.048), marijuana use dependence (p < 0.001), and major depression episode (MDE) symptoms (p < 0.001) were significantly associated with receiving opioid MAT status, respectively. Of those who had received opioid MAT, the majority were aged 26–34 years (0.68%), non-Hispanic Whites (0.43%), with some college credit but no degree (0.52%), less than $20,000 total family income (0.96%), and other employment status (0.55%). Additionally, among those who had received opioid MAT, 0.57% were bisexuals, 1.07% were lesbians/gays, 0.65% had alcohol use dependence, 2.32% had marijuana use dependence, and 1.59% experienced MDE symptoms.

### Multivariable logistic regression analysis

The weighted multivariable logistic regression analysis of the factors associated with receiving opioid MAT are presented in Table 2. People aged 26–34 (AOR = 9.77, 95% CI = 4.74, 20.17) and 35–49 (AOR = 5.61, 95% CI = 2.82, 11.18) years had higher odds of having received opioid MAT compared to those aged 18–25 years. Compared to females, males had higher odds of having received opioid MAT (AOR = 1.61, 95% CI = 1.10, 2.36). Non-Hispanic Black/African Americans (AOR = 0.26, 95% CI = 0.12, 0.59), and Hispanics (AOR = 0.13, 95% CI = 0.03, 0.58) had lower odds of having received opioid MAT compared to non-Hispanic Whites. Lesbians/gays had higher odds of having received opioid MAT (AOR = 3.43, 95% CI = 1.42, 8.25) compared to heterosexuals. Those who completed high school diploma/GED (AOR = 3.33, 95% CI = 1.41, 7.83), some college credit but no degree (AOR = 4.15, 95% CI = 1.56, 11.02), or associate's degree (AOR = 3.18, 95% CI = 1.04, 9.71) had higher odds of having received opioid MAT relative to those who completed college degree or higher. The odds of having received opioid MAT was lower for people with total family annual income of $20,000 - $49,999 (AOR = 0.40, 95% CI = 0.21, 0.76), $50,000 - $74,999 (AOR = 0.23, 95% CI = 0.12, 0.43), or $75,000 or more (AOR = 0.28, 95% CI = 0.14, 0.57) relative to those who had less than $20,000. The odds were higher for people with other employment status (AOR = 2.64, 95% CI = 1.59, 4.39) or unemployed status (AOR = 5.32, 95% CI = 2.19, 12.90) versus employed full-time; marijuana use dependence (AOR = 3.44, 95% CI = 1.47, 8.06) versus no marijuana use dependence; and MDE symptoms (AOR = 5.22, 95% CI = 3.46, 7.89) versus no MDE symptoms.

Table 3 presents the results of the weighted odds of receiving opioid MAT among individuals diagnosed with opioid use disorder symptoms. Males had higher odds of receiving opioid MAT (AOR = 2.39, 95% CI = 1.13, 5.04) compared to females. The odds were lower for other racial/ethnic individuals (AOR = 0.04, 95% CI = 0.01, 0.46) than for non-Hispanic White individuals; and for individuals with total family annual income of $50,000 - $74,999 (AOR = 0.20, 95% CI = 0.05, 0.74), or $75,000 or more (AOR = 0.28, 95% CI = 0.14, 0.57) compared to those who had less than $20,000. The odds were higher for individuals who were unemployed (AOR = 3.56, 95% CI = 1.30, 9.76) relative to those who were employed full-time.

## Discussion

The present analysis aimed to understand the prevalence and associations of opioid medication-assisted treatment (MAT) use among different high-risk groups using opioids or experiencing opioid use disorder (OUD) and mental health disorder symptoms. These high-risk groups are based on sexual identity, major depressive episode (MDE), alcohol dependence, and marijuana use dependence. Our study was the first to investigate the role of sexual identity in opioid MAT use within a U.S. representative sample. Our findings confirmed our hypothesis that sexual identity, MDE symptoms, and dependence on alcohol or marijuana use would be associated with opioid MAT use. Furthermore, MDE, dependence on alcohol or marijuana use, age, race/ethnicity, level of education, total family income, and employment status were associated with receiving opioid MAT. Younger adults and racial/ethnic minorities were less likely to receive opioid MAT, while those with a lower level of education, less income, unemployed, and MDE and marijuana use dependence symptoms were more likely to use opioid MAT.

MAT expansion to racial and ethnic minority persons may be a critical step in reducing and preventing these disorders' burden among underserved populations. White adults were more likely to receive a prescription to treat OUD than Black, Hispanic, and Native-Hawaiian/Pacific Islander/Asian American populations who also had OUD. Future research should explore the reasons for this disparity and develop, implement, and evaluate interventions that increase MAT use among racial and ethnic minority persons.

Being younger, living with a mental health disorder, having alcohol or marijuana dependence, and being in lower socioeconomic status were found to impede MAT use. Other researchers have reported that persons with lower socioeconomic status, mental health disorders, or alcohol or marijuana use disorders are more likely to have an OUD and could benefit from MAT use [33, 34]. While there are interventions for persons living with comorbid mental health and substance use disorders. There is a need for interventionists to adapt these interventions for persons who might benefit from MAT. The Substance Abuse and Mental Health Services Administration (SAMHSA) maintains a list of evidence-based interventions (see https://www.samhsa.gov/resource-search/ebp).

Lesbian, gay, or bisexual persons were more likely to use MAT than those identified as heterosexual [10– 13]. This positive finding suggests that MAT is an acceptable treatment option for sexual minority persons. As OUD is common among sexual minority persons, healthcare providers serving sexual minority persons are encouraged to screen patients for OUD and recommend MAT as part of the treatment and recovery plan. Due to a small sample of sexual minority persons, we combined lesbian, gay, and bisexual persons as a monolithic group. However, datasets with large sexual minority samples have identified differences in substance use patterns between subpopulations of sexual minority persons [35–38]. Future researchers with large samples of sexual minority individuals should examine intragroup differences in MAT use.

We found that males were more likely to receive MAT than females. These findings are consistent with previous studies on sex differences in MAT use [39, 40]. This sex disparity in MAT use could be due to stigma and lack of social support encountered in treatment and treatment success, which are more predominant among females than males [39, 41]. Females, in general, are more likely to use substances and experience disorders of mental health and substance use than males [39, 42–44], which could impede their substance use disorder treatments, including MAT use. There is an urgent need for interventions that address the unique concerns of women with an OUD to increase MAT initiation and maintenance.

Our study used data from the NSDUH, a standard and validated survey used widely across the U.S. to quantify substance use and its disorders. The use of this dataset allowed for a larger, more heterogeneous sample size for the analysis that reflected current U.S. racial and ethnic characteristics. Compared to a previous NSDUH study of OUD, the present study had a more diverse sample [25]. Moreover, our study adjusted for various sociodemographic factors as covariates for opioid MAT using a weighted multivariable logistic regression analysis. Despite these strengths, the study was not without limitations. The analysis was based on a cross-sectional survey susceptible to recall bias, given the dificulty of recalling past experiences. Due to the study's cross-sectional nature, we were also unable to make causal inferences between specific risk factors and respondents seeking MAT. As the NSDUH sample is representative of the general population, research with larger sexual minority samples is needed.

This manuscript is one of the first to report MAT use among sexual minority persons. While there is a need for additional research to understand intragroup differences among sexual minority persons, the higher use of MAT among sexual minority persons than among heterosexual persons presents an opportunity. There is an opportunity to capitalize on the acceptability of MAT to develop tailored interventions and reduce the incidence of OUD among sexual minority persons.

## Figures and Tables

**Table 1:** Prevalence of receiving opioid MAT across participants’ sociodemographic characteristics, dependence on alcohol and marijuana use, and MDE symptoms: A weighted sample of U.S. adults (N = 38,841), NSDUSH 2019

	Overall **N (%)**	Did not receive opioid medication-assisted treatment **n (%)**	Received opioid medication-assisted treatment **n (%)**	*p*-value
	38,841 (100%)	38,694 (99.69)	147 (0.31)	
**Age:**				< .001
18–25	14,226 (17.08)	14,200 (99.84)	26 (0.16)	
26–34	8,601 (20.41)	8,540 (99.32)	61 (0.68)	
35–49	11,134 (30.83)	11,083 (99.65)	51 (0.35)	
50–64	4,880 (31.68)	4,871 (99.89)	9 (0.11)	
**Sex:**				.2401
Male	18,176 (49.18)	18,099 (99.66)	77 (0.34)	
Female	20,665 (50.83)	20,595 (99.72)	70 (0.28)	
**Race/Ethnicity:**				.004
Non-Hispanic White	22,250 (59.66)	22,124 (99.57)	126 (0.43)	
Non-Hispanic	5,095 (12.68)	5,086 (99.83)	9 (0.17)	
Black/African				
American				
Hispanic	7,375 (18.52)	7,369 (99.93)	6 (0.07)	
Other race	4,121 (9.14)	4,115 (99.81)	6 (0.19)	
**Sexual identity**				.002
Heterosexual	34,455 (92.88)	34,327 (99.72)	128 (0.28)	
Lesbian or Gay	946 (2.32)	938 (98.93)	8 (1.07)	
Bisexual	2,614 (4.80)	2,603 (99.43)	11 (0.57)	
**Level of education completed:**				< .001
Twelfth grade or less grade	4,674 (11.54)	4,652 (99.71)	22 (0.29)	
High School diploma/GED	10,356 (23.59)	10,306 (99.59)	50 (0.41)	
Some college credit but no degree	9,658 (22.14)	9,603 (99.48)	55 (0.52)	
Associate's degree	3,724 (9.85)	3,712 (99.66)	12 (0.34)	
College graduate or higher	10,429 (32.89)	10,421 (99.91)	8 (0.09)	
**Total family income:**				< .001
Less than $20,000	7,157 (14.60)	7,093 (99.04)	64 (0.96)	
$20,000 - $49,999	11,557 (26.58)	11,512 (99.68)	45 (0.32)	
$50,000 - $74,999	6,135 (15.65)	6,117 (99.84)	18 (0.16)	
$75,000 or more	13,992 (43.17)	13,972 (99.86)	20 (0.14)	
**Employment status**				< .001
Employed full time	21,829 (59.94)	21,776 (99.85)	53 (0.15)	
Employed part time	6,214 (13.38)	6,197 (99.67)	17 (0.33)	
Unemployed	2,448 (4.70)	2,426 (98.80)	22 (0.20)	
Other	8,350 (21.97)	8,295 (99.45)	55 (0.55)	
**Past year alcohol use dependence:**				0.048
No	37,178	37,043 (99.70)	135 (0.30)	
Yes	1,663 (3.73)	1,651 (99.35)	12 (0.65)	
**Past year marijuana use dependence:**				< .001
No	37,978 (98.58)	37,845 (99.72)	133 (0.28)	
Yes	863 (1.42)	849 (97.68)	14 (2.32)	
**Past year MDE symptoms:**				< .001
No	33,997 (90.87)	33,911 (99.81)	86 (0.19)	
Yes	4,335 (9.13)	4,274 (98.41)	61 (1.59)	

Data Source: National Survey on Drug Use and Health (NSDUH) 2019.

1. *Weighted N =* 197,554,210 *and unweighted N* = 38,841

2. Statistical significance at p < 0.05.

3. All p-values are based on chi-square tests for the categorical variables.

4. MDE = Major Depressive Episode.

5. MDE has 509 (unweighted = 1.31% and weighted = 1.24%) and sexual identity has 826 (unweighted = 2.13% and weighted = 2.25%) missing observation out of 38,841.

**Table 2:** Adjusted odds ratios of receiving opioid MAT associated with sociodemographic characteristics, dependence on alcohol and marijuana use, and MDE symptoms.

	AOR	95% CI	
**Age:**			
18–25	Ref	-	
26–34	9.77***	(4.74, 20.17)	
35–49	5.61***	(2.82, 11.18)	
50–64	1.37	(0.50, 3.77)	
**Sex:**			
Female	Ref	-	
Male	1.61**	(1.10, 2.36)	
**Race/Ethnicity:**			
Non-Hispanic White	Ref	-	
Non-Hispanic Black/African American	0.26**	(0.12, 0.59)	
Hispanic	0.13**	(0.03, 0.58)	
Other race	0.47	(0.16, 1.39)	
**Sexual identity:**			
Heterosexual (i.e., straight)	Ref	-	
Lesbian or Gay	3.43**	(1.42, 8.25)	
Bisexual	0.90	(0.44, 1.85)	
**Level of education completed:**			
College graduate or higher	Ref	-	
Twelfth grade or less grade	2.55	(0.82, 7.92)	
High School diploma/GED	3.33**	(1.41, 7.83)	
Some college credit but no degree	4.15**	(1.56, 11.02)	
Associate's degree	3.18*	(1.04, 9.71)	
**Total family income:**			
Less than $20,000	Ref		-
$20,000 - $49,999	0.40**		(0.21, 0.76)
$50,000 - $74,999	0.23***		(0.12, 0.43)
$75,000 or more	0.28***		(0.14, 0.57)
**Employment status:**			
Employed full time	Ref		-
Employed part-time	1.95		(0.84, 4.52)
Unemployed	5.32***		(2.19, 12.90)
Other	2.64***		(1.59, 4.39)
**Past year alcohol use dependence:**			
No	Ref		-
Yes	0.85		(0.36, 2.02)
**Past year marijuana use dependence:**			
No	Ref		-
Yes	3.44**		(1.47, 8.06)
**Past year MDE symptoms:**			
No	Ref		-
Yes	5.22***		(3.46, 7.89)

*Note: Weighted N =* 191,664,622 and *unweighted N =* 37,506

AOR = Adjusted odds ratio. 95% CI = 95% confidence interval.

* p < 0.05,

** p < 0.01, and

*** p < 0.001,

Ref. = Reference group.

MDE = Major Depressive Episode.

**Table 3:** Sociodemographic characteristics, dependence on alcohol and marijuana use, and MDE symptoms associated with the odds of receiving opioid MAT among individuals diagnosed with opioid use disorder

		AOR	95% CI
**Age:**			
18–25	Ref		-
26–34	1.74		(0.64, 4.78)
35–49	1.02		(0.36, 2.93)
50–64	0.20		(0.04, 1.01)
**Sex:**			
Female	Ref		-
Male	2.39*		(1.13, 5.04)
**Race/Ethnicity:**			
Non-Hispanic White	Ref		-
Non-Hispanic Black/African American	0.82		(0.25, 2.74)
Hispanic	0.58		(0.15, 2.23)
Other race	0.04**		(0.01, 0.46)
**Sexual identity:**			
Heterosexual (i.e., straight)	Ref		-
Lesbian/Gay or Bisexual	2.24		(0.70, 7.13)
**Level of education completed:**			
College graduate or higher	Ref		-
Twelfth grade or less grade	0.16		(0.03, 1.07)
High School diploma/GED	0.49		(0.13, 1.83)
Some college credit but no degree	0.67		(0.14, 3.21)
Associate's degree	1.10		(0.20, 5.97)
**Total family income:**			
Less than $20,000		Ref	-
$20,000 - $49,999		0.67	(0.29, 1.59)
$50,000 - $74,999		0.20**	(0.05, 0.74)
$75,000 or more		0.51	(0.17, 1.48)
**Employment status:**			
Employed full time		Ref	-
Employed part-time		1.39	(0.45, 4.26)
Unemployed		3.56**	(1.30, 9.76)
Other		2.19	(0.89, 5.39)
**Past year alcohol use dependence:**			
No		Ref	-
Yes		0.55	(0.17, 1.79)
**Past year marijuana use dependence:**			
No		Ref	-
Yes		1.68	(0.55, 5.07)
**Past year MDE symptoms:**			
No		Ref	-
Yes		1.46	(0.68, 3.13)

*Note: Weighted N =* 1,386,436 *unweighted N =* 302

AOR = Adjusted odds ratio. 95% CI = 95% confidence interval.

* p < 0.05,

** p < 0.01, and

*** p < 0.001,

Ref. = Reference group.

MDE = Major Depressive Episode.
